# Supplementary material for: Computational design of peptides to target NaV1.7 channel with high potency and selectivity for the treatment of pain
Source: eLife. 2022 Dec 28;11:e81727. doi: 10.7554/eLife.81727 (PMC9831606; doi:10.7554/eLife.81727)
Supplement: Figure 4—figure supplement 1—source data 1. [file elife-81727-fig4-figsupp1-data1.docx]

|  | **Nav1.7** | **Nav1.1** | **Nav1.2** | **Nav1.3** | **Nav1.4** | **Nav1.5** | **Nav1.6** | **Nav1.8** | **Nav1.9** |
| --- | --- | --- | --- | --- | --- | --- | --- | --- | --- |
| **PTx2-3127** | 6.9 | 16,970 | 5,040 | 20,040 | 11,530 | 137,090 | 608 | >150,000 | >150,00 |
| **PTx2-3128** | 5.0 | 3,300 | 570 | 23,000 | 22,000 | 34,000 | 358 | 10,000 | 8,000 |
