# Supplementary material for: Computational design of peptides to target NaV1.7 channel with high potency and selectivity for the treatment of pain
Source: eLife. 2022 Dec 28;11:e81727. doi: 10.7554/eLife.81727 (PMC9831606; doi:10.7554/eLife.81727)
Supplement: Figure 5—figure supplement 1—source data 1. [file elife-81727-fig5-figsupp1-data1.docx]

|  | Nav1.7 | Nav1.1 | Nav1.2 | Nav1.3 | Nav1.4 | Nav1.5 | Nav1.6 | Nav1.8 | Nav1.9 |
| --- | --- | --- | --- | --- | --- | --- | --- | --- | --- |
| PTx2-3258 | 3.8 | 5,013 | 3,399 | 14,093 | 8,877 | 38,315 | 382 | 43,079 | 59,443 |
